# Exploring the relationship of cognitive function with and without COVID-19 recovered schizophrenic patients

**DOI:** 10.3389/fpubh.2023.1306132

**Published:** 2024-01-03

**Authors:** Anam Mehmood, Ali Madi Almajwal, Abdullah Addas, Falak Zeb, Iftikhar Alam, Bismillah Sehar

**Affiliations:** ^1^Department of Psychiatry, The Second Xiangya Hospital, Central South University, Changsha, Hunan, China; ^2^Department of Community Health Sciences, College of Applied Medical Sciences, King Saud University, Riyadh, Saudi Arabia; ^3^Department of Civil Engineering, College of Engineering, Prince Sattam Bin Abdulaziz University, Al-Kharj, Saudi Arabia; ^4^Landscape Architecture Department, Faculty of Architecture and Planning, King Abdulaziz University, Jeddah, Saudi Arabia; ^5^Research Institute of Medical and Health Sciences, University of Sharjah, Sharjah, United Arab Emirates; ^6^Department of Human Nutrition and Dietetics, Bacha Khan University Charsadda, KPK, Pakistan; ^7^Department of Health and Social Sciences, University of Bedfordshire, Luton, United Kingdom

**Keywords:** COVID-19, cognitive performance, schizophrenia, BACS, PANSS

## Abstract

**Background:**

The Coronavirus disease 2019 (COVID-19) is linked to the deterioration of cognitive function among individuals suffering from schizophrenia. The purpose of this study was to compare the cognitive performance of schizophrenic patients before and after COVID-19.

**Methods:**

A longitudinal cohort study involving a sample of 219 individuals diagnosed with schizophrenia was enrolled between June 2022 and May 2023. The participants were split into two groups infected with COVID-19 (*n* = 165) and not infected with COVID-19 (*n* = 54). The data were gathered via a questionnaire on demographic characteristics, the Brief Assessment of Cognition in Schizophrenia (BACS), the Positive and Negative Syndrome Scale (PANSS), the Calgary Depression Scale for Schizophrenia (CDSS), the Activities of Daily Living (ADL) scale, and the Insomnia Severity Index (ISI).

**Results:**

The repeated-measures ANOVA showed that Among patients diagnosed with COVID-19, there was a deterioration in global cognitive function (before COVID-19 = −2.45 vs. after COVID-19 = −3.02, *p* = 0.007), working memory (before COVID-19 = −2.76 vs. after COVID-19 = −3.34, *p* < 0.00 1), motor speed (before COVID-19 = −1.64 vs. after COVID-19 = −2.12, *p* < 0.001), attention and speed of information processing (before COVID-19 = −1.93 vs. after COVID-19 = −1.16, *p* = 0.008). multi-variable analysis showed that several factors as having a secondary grade of education (*β* = 0.434), experiencing insomnia (*β* = 0.411)and the interaction between COVID-19 diagnosis and cognition at baseline (*β* = 0.796) were significantly associated with cognitive deficits. At the same time, no significant associations were found between global cognition and clinical symptoms, autonomy, or depression (*p* > 0.05).

**Conclusion:**

The COVID-19 pandemic has significantly impacted various cognitive functions, such as verbal memory, working memory, and global cognition. Insomnia has been identified as the predominant determinant of cognitive impairment, alongside the confirmation of a COVID-19 diagnosis. Additional research is imperative to elucidate the diversification of cognitive functionality observed in individuals diagnosed with schizophrenia who have acquired COVID-19.

## Introduction

COVID-19 is primarily a respiratory illness with the potential to affect other organs, most notably the brain (1). It is caused by SARS-COV-2, a coronavirus that causes severe acute respiratory syndrome.SARS-COV-2 may target the central nervous system, resulting in severe neurological dysfunction or interfering with peripheral nerves and causing sensory loss, skeletal muscle pain, dizziness, decreased consciousness, acute cerebrovascular disease, and epilepsy (2, 3). Additionally, neuropsychiatric symptoms, including suicide, sleeplessness, mood changes, psychosis, encephalopathy, and cognitive impairment, may manifest (4, 5). COVID-19’s particular psychophysiology mechanisms and the etiology of its psychiatric, neurological, and cognitive dysfunctions have not yet been established, although they are likely complex (6). The Coronavirus disease 2019 (COVID-19) pandemic has aroused concerns about its possible effects on mental health and being an imminent risk to global public health. Severe mental illness makes people more susceptible to public health emergencies like the COVID-19 worldwide pandemic (7). Public health actions, unable to purchase medication or seek medical attention promptly due to isolation and limited transportation, and measures taken to halt the spread of COVID-19 may exacerbate the symptoms of those who already have psychotic illnesses or raise the likelihood of people developing new psychotic episodes (8). This trend is consistent with what we observe in those who struggle with anxiety, addiction, and depression (9).

Post-COVID-19 cognitive deterioration is a significant clinical feature flick of the disease and can have long-lasting effects (10). A COVID-19 infection can lead to a decline in cognitive performance, resulting in liabilities in various areas such as working memory, set-shifting, verbal learning, divided attention, motor speed, and executive functions (11, 12). Insomnia can also be a symptom of cognitive deterioration, and some patients may experience severe difficulties in their daily lives. Recent researchers reported that a considerable number of patients experience persistent cognitive impairments, such as memory problems, difficulty concentrating, and insomnia, even after recovering from COVID-19 (13). Individuals who have recovered from COVID-19 exhibit obvious impairments in attention, executive function, and memory, with reports of delirium, systemic inflammation, and evidence of neurotropism (11, 14). The differences in the incidence and severity of cognitive dysfunction in individuals are likely to be multifaceted, depending on various biological, social, and economic factors (15). In the long haul, there is also trepidation that these cognitive deficiencies may accelerate the emergence of neurodegenerative diseases like Alzheimer’s disease (16). A recent meta-analysis reported that mental health complications were concomitant with higher than usual COVID-19-related deaths (17).

The COVID-19 epidemic has had an immense impact on populations, particularly those who are more vulnerable, including those with schizophrenia (10). Schizophrenia stands out as a complex condition characterized by a range of cognitive impairments among various mental health disorders field (18). Schizophrenia is a degenerative and persistent mental health condition characterized by cognitive deficits like disrupted working memory and executive function, negative psychotic symptoms like anhedonia and antisocially, and positive psychotic symptoms like delusions and hallucinations (1). Schizophrenic patients are particularly likely to propagate the virus because they have difficulty adopting the preventative measures indicated to avert infection because of an immune-related mechanism and psychotic symptoms (19). Antipsychotic treatments also affect cognitive impairment in a lot of schizophrenic patients (20). Cognitive deterioration is a key distinguishing feature of schizophrenia that emerges early in the illness and may indicate medication side effects (21). Memory, motor skills, attention, social cognition, and executive function are just a few of the cognitive abilities that are affected in people with schizophrenia (18).

The COVID-19 epidemic has undoubtedly had a substantial consequence in Pakistan, and there is a pressing need to investigate the cognitive function of schizophrenic patients who have recovered from the virus. Understanding the potential effects of COVID-19 on this population’s cognitive abilities can provide valuable insights into the underlying mechanisms and guide effective interventions. This study is intended to clarify the intricate relationship between COVID-19 and cognitive function in schizophrenic patients in Pakistan. By examining the cognitive profiles of individuals who have experienced both schizophrenia and COVID-19, researchers can identify potential risk factors that may influence cognitive outcomes. This exploration of cognitive function in schizophrenic patients recovered from COVID-19 holds immense significance for several reasons. Firstly, it can impart our understanding of the prolonged impact of COVID-19 on mental health, specifically for individuals with pre-existing psychiatric conditions. Secondly, it may assist in establishing focused interventions and treatment plans to mitigate cognitive impairments in this vulnerable populationCOVID-19 is primarily a respiratory illness with the potential to affect other organs, most notably the brain (1). It is due to severe acute respiratory syndrome coronavirus-2(SARS-COV-2). SARS-COV-2 may target the central nervous system, which could result in severe neurological dysfunction or interfere with peripheral nerves, causing sensory loss, skeletal muscle pain, dizziness, decreased consciousness, acute cerebrovascular disease, and epilepsy (2, 3).

Additionally, neuropsychiatric symptoms, including suicide, sleeplessness, mood changes, psychosis, encephalopathy, and cognitive impairment, may manifest (4, 5). COVID-19’s particular psychophysiology mechanisms and the etiology of its psychiatric, neurological, and cognitive dysfunctions have not yet been established, although they are likely complex (6). The COVID-19 pandemic has not only posed a significant threat to global public health but has also raised concerns about its potential impact on mental health. Severe mental illness makes people more susceptible to public health emergencies, such as the COVID-19 globe-wide pandemic (7). Public health actions, the inability to purchase medication or seek medical attention promptly due to isolation and limited transportation, and measures taken to halt the spread of COVID-19 may exacerbate the symptoms of those with psychotic illnesses or raise the likelihood of people developing new psychotic episodes (8). This pattern parallels that observed in individuals with anxiety, substance use, and depression (9).

Post-COVID-19 cognitive impairment is a significant clinical feature of the disease and can have long-lasting effects (10). A COVID-19 infection can lead to a decline in cognitive performance, resulting in deficits in various areas such as verbal learning, working memory, set-shifting, divided attention, motor speed, and executive functions (11, 12). Additionally, insomnia can also be a symptom of cognitive deterioration, and some patients, may experience severe difficulties in their daily lives. Recent studies have reported that a considerable number of patients experience persistent cognitive impairments, such as memory problems, difficulty concentrating, and insomnia, even after recovering from COVID-19 (13). Individuals who have recovered from COVID-19 exhibit obvious impairments in attention, executive function, and memory, with reports of delirium, systemic inflammation, and evidence of neurotropism (11, 14). Differences in the incidence and severity of cognitive dysfunction in individuals will likely be multifaceted, depending on various biological, social, and economic factors (15). There is also concern that these cognitive impairments could intensify the onset of neurodegenerative disorders such as Alzheimer’s disease in the long run (16). A recent systematic review and meta-analysis of 16 observational studies in seven countries showed that mental health disorders were associated with increased COVID-19-related mortality (17).

The COVID-19 pandemic has immensely impacted populations, particularly those more vulnerable, including those with schizophrenia (10). Schizophrenia is a chronic and disabling mental health condition associated with positive psychotic symptoms such as delusions and hallucinations, negative psychotic symptoms such as anhedonia, and sociality, and cognitive symptoms such as impaired working memory and executive function (1). schizophrenic patients are prone to spreading the virus because of their difficulty adhering to the preventative measures recommended owing to an immune-related mechanism and psychotic symptoms (19). Symptoms of psychotic treatments also contribute to cognitive impairment in many patients with schizophrenia (20).

Cognitive impairment is a crucial characteristic of schizophrenia that manifests at the beginning of the illness and may be related to medicinal side effects (21). In individuals with schizophrenia, various cognitive functions such as motor skills, memory, executive function, social cognition, and attention are impaired (18). The COVID-19 pandemic has devastating consequences in Pakistan, and there is a pressing need to investigate the cognitive function of schizophrenic patients who have recovered from the virus. Understanding the potential effects of COVID-19 on this population’s cognitive abilities can provide valuable insights into the underlying mechanisms and guide effective interventions. This study aims to shed light on the complex relationship between COVID-19 and cognitive function in schizophrenic patients in Pakistan. By examining the cognitive profiles of individuals who have experienced both schizophrenia and COVID-19, researchers can identify potential risk factors that may influence cognitive outcomes. This exploration of cognitive function in schizophrenic patients recovered from COVID-19 holds immense significance for several reasons. Firstly, it can bring to our understanding the long-term consequences of COVID-19 on mental health, specifically for individuals with preexisting psychiatric conditions. Secondly, it can assist in developing targeted interventions and treatment schemes to classify cognitive impairments in this vulnerable population. Lastly, this research can provide valuable insights into the broader field of neuropsychiatry, offering a unique perspective on the complex interplay between infectious diseases, mental health, and cognitive functioning. By delving into this unexplored territory, this study seeks to unravel the mysteries surrounding the cognitive function of schizophrenic patients who have recovered from COVID-19 in Pakistan and support the expansion of knowledge on the impact of COVID-19 on mental health. Ultimately, the findings of this research can pave the way for improved care and support for individuals with schizophrenia.

Lastly, his research can provide valuable insights into the broader field of neuropsychiatry, offering a unique perspective on the complex interplay between infectious diseases, mental health, and cognitive functioning. By delving into this unexplored territory, this study seeks to unravel the mysteries surrounding the cognitive function of schizophrenic patients who have recovered from COVID-19 in Pakistan and contribute to the growing body of knowledge on the impact of COVID-19 on mental health. Ultimately, the findings of this research can pave the way for improved care and support for individuals with schizophrenia.

## Methods

### Study context and subjects

A prospective cohort research study was carried out among 219 individuals suffering from schizophrenia receiving treatment at Mayo Hospital in Lahore, Pakistan. The study was carried out from June 2022 to May 2023 to investigate the impact of COVID-19 on the cognitive function of individuals with schizophrenia. The participants were divided into two groups: those with a COVID-19 diagnosis (*n* = 165) and those without a COVID-19 diagnosis (*n* = 54). To represent the highest research standards, patients were monitored for an average of 6.52 ± 1.94 months after contracting COVID-19 in the hospital. Cognitive assessments were conducted in two phases: the first was between June and December 2022, and the second was between March and May 2023. Strict inclusion and exclusion criteria were applied to ensure accurate results. The study was strictly restricted to individuals between 18 and 60 who had previously had a cognitive test, finished at least their first year of school, and fulfilled the DSM-5 criteria for schizophrenia. Patients with conditions that could potentially influence cognitive performance, such as head injury, drug abuse, or neurological disorders, and do not meet DMS-5 criteria for schizophrenia were excluded from the study. Positive real-time reverse transcriptase-polymerase chain reaction (RT-PCR) assays, which are well recognized as a diagnostic tool for identifying SARS-CoV-2 virus RNA in clinical specimens, have been deployed to confirm the diagnosis of COVID-19 (22).

Overall, this study was conducted with the utmost professionalism and adherence to research standards, aiming to provide evidence-based insights into the impact of COVID-19 on cognitive function among individuals with schizophrenia.

### Ethics statement

The study obtained approval from the Research Ethics Committee of Mayo Hospital in Lahore, Pakistan, and all participants provided informed consent. All collected data were kept anonymous for confidentiality. Entirely methods were performed following the relevant guidelines and regulations.

### Measures

The questionnaire was presented in Urdu, which is the language of the nation of Pakistan. The questionnaire’s first section inquired about demographic details such as gender, age, BMI, education level, economic situation, marital status, family history of mental illness, length of disease, and number of hospitalizations. The World Health Organization (WHO) classification system, which comprises asymptomatic, mild, moderate, severe, and serious cases, was adopted to classify the severity of COVID-19and the severity of COVID-19 was categorized using the World Health Organization (WHO) categorization system, which includes asymptomatic, mild, moderate, severe, and serious instances (23).

Pervasive post-COVID-19 symptoms were transcribed using a checklist. Medication regimens were obtained by reviewing the participant’s medical records, and the Andersen approach for antipsychotics was used to calculate the chlorpromazine dose equivalent (24). The Anticholinergic Drug Scale (ADS) was utilized to rank anticholinergic medications, with each medication allotted a numerical value Field (25). The total ADS score was calculated by summing the values of all scheduled drugs used by each participant. The second section of the questionnaire included the Brief Assessment of Cognition in Schizophrenia (BACS), the Positive and Negative Syndrome Scale (PANSS), the Calgary Depression Scale for Schizophrenia (CDSS), the Activities of Daily Living (ADL) scale, and the Insomnia Severity Index (ISI). By employing these measures, we aimed to assess the cognitive function comprehensively and entailed factors among individuals with schizophrenia who have recovered from COVID-19.

### Brief assessment of cognition in schizophrenia

Brief Assessment of Cognition in Schizophrenia (BACS) is a neuropsychological tool specifically designed to assess and monitor the cognitive functioning of individuals with schizophrenia (26). It consists of six subscales that measure verbal memory, psychomotor function, attention, working memory, verbal fluency, executive function, and information processing speed. In our research, the measure demonstrated good reliability with a Cronbach’s α of 0.867 at baseline and a Cronbach’s α of 0.832 after the COVID-19 infection.

### The positive and negative syndrome scale

Positive and Negative Syndrome Scale (PANSS) is a widely used survey tool for assessing symptoms in individuals with schizophrenia (27). It consists of 30 items, each scored on a scale from 1 to 7, indicating the absence of the severity of symptoms. The total score reflects the overall intensity of symptoms, with higher scores indicating greater symptom severity. In our research, the PANSS demonstrated good reliability, with a Cronbach’s α of 0.854 at baseline and 0.778 after the onset of COVID-19.

### Calgary depression scale for schizophrenia

Calgary Depression Scale for Schizophrenia (CDSS)is a validated tool designed to assess and monitor depressive symptoms in individuals with schizophrenia (28). It consists of nine items that evaluate various aspects of depression, such as hopelessness, self-depreciation, guilt, and suicidal thoughts. Additionally, one item assesses observable depressive traits. The CDSS score reflects the severity of depressive symptoms, with higher scores indicating greater intensity. In our research, the CDSS demonstrated good reliability, with a Cronbach’s α of 0.808 at baseline and 0.799 after the onset of COVID-19.

### Activities of daily living

Activities of Daily Living (ADL)is an assessment tool used to objectively evaluate an individual’s ability to perform essential activities of daily living (29). It is commonly utilized in research and clinical settings and comprises six items that assess functional capabilities in personal hygiene, dressing, toileting, mobility, continence, and feeding. The total score is calculated by summing up the scores of all six items, with higher scores indicating a greater level of functional independence in daily life. In our research, the measure demonstrated good reliability, with a Cronbach’s α of 0.738 at baseline and 0.729 after the onset of COVID-19.

### Insomnia severity index

Insomnia Severity Index (ISI) is a widely accepted measure used to assess the severity of perceived insomnia symptoms (30). It consists of seven items that evaluate the severity of difficulties in falling asleep, satisfaction with current sleep patterns, interference with daytime functioning, and distress levels related to sleep problems. The total score is obtained by summing the scores of all seven items with a maximum score of 28. Our current research found that the ISI exhibited good reliability with a Cronbach’s α of 0.867 at baseline and 0.829 after the onset of COVID-19. The reliability analysis of all the constructs have been shown in Table 1.

**Table 1:** Reliability analysis.

	Cronbach’s alpha	
	*At baseline*	*After COVID-19*
BACS	0.867	0.832
PANSS	0.854	0.778
CDSS	0.808	0.799
ADL	0.738	0.729
ISI	0.867	0.829

### Statistical analysis

To provide a comprehensive understanding of the data, a descriptive analysis was performed. Categorical variables were expressed in absolute frequencies and percentages, while quantitative variables were presented using means and standard deviations. The composite standardized z-score of the Brief Assessment of Cognition in Schizophrenia (BACS)was computed by averaging the total score from the mean total score of the control group (31). This control group was chosen from the same database, ensuring consistency and validity of the results. Furthermore, a normative cognitive change was calculated based on the group of patient’s normative cognitive change was computed based on group of patients uninfected by COVID-19. This analysis aimed to look into the percentage of patients whose cognitive functioning declined compared to normative cognitive change. The formula used for patients without COVID-19 was(Y2-Y1)/SD, where Y1 represents the mean standardized composite score of BACS before COVID-19, Y2 refers to the standardized composite score of BACS after COVID-19, and SD represents the standard deviation of the BACS measurement from the pre-COVID-19 period. The resulting variation was 0.73, which served as the criterion value. BACS composite standardized score near zero indicates cognitive improvement, while scores less than 0.73 after COVID-19 indicate impaired cognition.

A repeated-measures ANOVA was conducted to evaluate the variation in the BACS total composite standardized score and sub-scores over time. This analysis considered factors of clinical symptoms, chlorpromazine equivalent dose, ADS score, and ISI score. The analysis was conducted for two groups of patients: those infected with COVID-19 and those who were not. General Linear Model Univariate analysis was conducted, with the fixed factor involved in the analysis being whether the individual was diagnosed with COVID-19. The analysis was adjusted for various factors, including age, gender, BMI, education level, clinical symptoms, depression, cognitive assessment score at baseline, duration between cognitive assessments, chlorpromazine equivalent dose, ADL and ADS score, and ISI score. The analysis was performed using SAS version 9.4(SAS Institute, Cary, NC), and statistical significance was considered at *p* < 0.05.

## Results

### Description of the characteristics of COVID-19 participants

The study comprised 219 participants with schizophrenia diagnoses, 165 (75.34%) of whom possessed a COVID-19 diagnosis. The average age of the participants with schizophrenia was 48.43 ± 6.89 years, and the majority were male (57.53%). The mean BMI of the participants was 21.9 ± 5.2, indicating an average body mass index within a healthy range. Regarding education, 49.77% of the participants had completed secondary education. Most participants (72.60%) reported low economic status and a significant proportion (77.17%) were single. Additionally, 10.5% of the participants reported a family history of mental health disorders. On average, the duration of illness for the participants was 14.65 ± 9.15 months, indicating a relatively long-term condition, and the participants had 5.45 ± 2.32 time’s hospitalizations, suggesting a history of recurrent hospital visits. In the total sample, 75.34% of individuals were diagnosed with COVID-19, with the majority (72.60%) experiencing mild symptoms. The average duration between SARS-CoV-2 infection and the subsequent cognitive evaluation was 7.21 ± 2.51 months. Table 2 summarizes the participants’ descriptive characteristics.

**Table 2:** Descriptive statistics of the study participants.

Demographics	Frequency (%)
Age	48.43 ± 6.89
Gender	
Male	126(57.53%)
Female	93(42.47%)
BMI (kg/m2)	21.9 ± 5.2
Educational level	
Primary	65(29.68%)
Middle	28(12.79%)
Secondary	109(49.77%)
University	17(7.76%)
Economic status	
High	36(16.44%)
Middle	24(10.96%)
Low	159(72.60%)
Marital status	
Single	169(77.17%)
Married	17(7.76%)
Separated	33(15.07%)
Family history of mental health disorders	
Yes	23(10.5%)
No	196(89.5%)
Duration of illness(months)	14.65 ± 9.15
Number of hospitalizations(average)	5.45 ± 2.32

### Cognitive variation before and following COVID-19 infection

Table 3 illustrates the findings addressing the impact of the COVID-19 diagnosis on cognitive function among all patients, regardless of their infection status. A normative cognitive change was determined by analyzing the variance in the BACS composite score; a significant decline in cognitive function was observed in 89.79% of cases who tested positive for COVID-19. Before the COVID-19 period, there was a notable difference in the BACS score between patients who experienced a major cognitive deterioration and those who did not (BACS score for the deteriorated group = −1.98, BACS score for the non-deteriorated group = −0.62, *p* < 0.001). Following the COVID-19 period, patients with a major cognitive decline exhibited a significantly higher cognitive deficit as compared to those without (BACS score for the deteriorated group = −2.69, BACS score for the non-deteriorated group = 0.73, *p* < 0.001). To further investigate the impact of COVID-19 on cognitive function, repeated measure ANOVA was conducted to compare cognitive performance before and after SARS-CoV-2 infection. Among patients diagnosed with COVID-19,there was a deterioration in global cognitive function (before COVID-19 = −2.45 vs. after COVID-19 = −3.02, *p* = 0.007), working memory (before COVID-19 = −2.76 vs. after COVID-19 = −3.34, *p* < 0.001), motor speed (before COVID-19 = −1.64 vs. after COVID-19 = −2.12, *p* < 0.001), attention and speed of information processing (before COVID-19 = −1.93 vs. after COVID-19 = −1.16, *p* = 0.008). However, it is worth noting that verbal memory improved after COVID-19 (before COVID-19 = −3.11 vs. after COVID-19 = −2.46, *p* < 0.001).

**Table 3:** ANOVA of the cognitive performance in individuals with schizophrenia before and after COVID-19 infection.

	Patients’ with COVID-19 (*n* = 165)			Patients’ with COVID-19 (*n* = 54)		
	Pre-COVID-19 period	Post-COVID-19 period	Value of *p*	Baseline period	Follow up period	Value of *p*
	Mean ± *SD* (95%CI)	Mean ± *SD* (95%CI)		Mean ± *SD* (95%CI)	Mean ± *SD* (95%CI)	
BACS (global score)	−2.45 ± 1.96 (−4.41;−0.49)	−3.02 ± 1.72 (−1.30;−4.74)	0.007	−3.19 ± 2.02 (−1.17;−5.21)	−2.61 ± 2.01 (−0.60;−4.62)	0.279
Verbal memory	−3.11 ± 1.84 (−1.27;−4.95)	−2.46 ± 1.78 (−4.24;−0.68)	<0.001	−3.62 ± 1.01 (−2.61;−4.63)	−2.96 ± 2.15 (−0.81;−4.84)	0.043
working memory	−2.76 ± 1.98 (−0.78;−4.74)	−3.34 ± 1.99 (−1.35;−5.33)	<0.001	−2.97 ± 2.23 (−0.74;−5.20)	−3.11 ± 1.98 (−1.13;−5.09)	0.483
Motor task	−1.64 ± 1.01 (−0.63;−2.65)	−2.12 ± 2.20 (0.08;−4.32)	<0.001	−1.85 ± 0.82 (−1.03;−2.67)	−2.82 ± 1.81 (−1.01;−4.63)	0.023
Verbal fluency	−2.46 ± 1.57 (−0.89;−4.03)	−2.23 ± 1.01 (−1.22;−3.24)	0.126	−3.35 ± 1.89 (−1.46;−5.24)	−3.68 ± 1.21 (−2.47;−4.89)	0.633
Attention and speed of information processing	−1.93 ± 0.81 (−1.12;−2.74)	−1.98 ± 1.23 (−0.75;−3.21)	0.006	−3.34 ± 1.67 (−1.67;−5.01)	−3.75 ± 1.58 (−2.17;−5.33)	0.329
Executive functioning	−3.36 ± 1.76 (−1.60;−5.12)	−3.89 ± 2.09 (−1.80;−5.98)	0.210	−3.11 ± 1.54 (−1.57;−4.65)	−3.33 ± 1.69 (−1.64;−5.02)	0.123
PANSS(Positive)	23.55 ± 9.22 (32.77;14.33)	19.31 ± 8.30 (27.61;11.01)	0.002	20.42 ± 7.56 (27.98;12.86)	18.65 ± 6.06 (24.71;−24.71)	0.023
PANSS(Negative)	16.22 ± 8.01 (24.23;8.21)	12.12 ± 7.11 (19.23;5.01)	0.003	17.56 ± 8.88 (26.44;8.68)	12.66 ± 7.09 (19.75;5.57)	0.034
PANSS (General psychopath- logy)	42.12 ± 14.38 (56.5;27.74)	33.11 ± 9.09 (42.2;24.02)	<0.001	44.45 ± 17.17 (61.62;27.28)	31.98 ± 10.09 (42.07;21.89)	0.021
CDSS (depression)	4.43 ± 3.98 (8.41;0.45)	4.32 ± 3.22 (7.54;1.1)	0.321	5.55 ± 4.33 (9.88;1.22)	5.93 ± 4.45 (10.38;1.48)	0.465
ADL	6.01 ± 0.99 (7;5.02)	5.99 ± 0.54 (6.53;5.45)	0.417	6.80 ± 0.84 (6.92;5.96)	5.93 ± 0.73 (6.66;5.2)	0.714
Chlorpromazine equivalent dose	1120.50 ± 942.24 (2062.74;178.26)	1124.38 ± 999.34 (2123.72;125.04)	0.791	1522.06 ± 1208.56 (2730.62;313.5)	1730.89 ± 1094.07 (2824.96;636.79)	0.059
ADS score	8.24 ± 3.08 (11.32;5.16)	8.11 ± 2.99 (11.1;5.12)	0.753	9.37 ± 3.01 (12.38;6.36)	8.29 ± 2.03 (10.32;6.26)	0.186
Insomnia severity index score	6.79 ± 0.99 (7.78;5.8)	6.85 ± 1.99 (8.57;4.86)	0.034	7.02 ± 1.43 (8.45;5.59)	7.22 ± 1.02 (8.24;6.2)	0.016

Significant level *p* < 0.05.

Conversely, verbal memory improved after COVID-19 (before COVID-19 = −3.62 vs. after COVID-19 = −2.96, *p* = 0.043). Among patients without COVID-19, the results indicated a decrease in motor speed after the COVID-19 period (before COVID-19 = −1.85 vs. after COVID-19 = −2.82, *p* = 0.023). Among patients diagnosed with COVID-19, there was a significant decline in clinical symptoms (PANSS positive, PANSS negative, and PANSS general psychopathology scores) and insomnia scores after the COVID-19 period. This suggests that these patients experienced an improvement in their clinical symptoms and sleep quality following their COVID-19 diagnosis. On the other hand, patients without COVID-19 also showed a decline in clinical signs (PANSS negative and PANSS general psychopathology scores), indicating an improvement in their overall psychiatric condition. However, this change was not statistically significant. There were no significant changes observed for ADS scores, depression, chlorpromazine equivalent dose, and insomnia before and after the COVID-19 period among both groups of patients. This suggests that these variables remained relatively stable and were not significantly affected by the COVID-19 diagnosis.

In summary, individuals diagnosed with COVID-19 showed a significant improvement in clinical symptoms and insomnia after the COVID-19 period, while patients without COVID-19 showed a non-significant decline in clinical signs. Other variables, such as depression, chlorpromazine equivalent dose, ADS scores, and insomnia, did not exhibit significant changes in either group.

### Multi-variable analysis

In terms of selecting global cognition after COVID-19 contagion as the dependent variable, t. The results of the multi-variable analysis showed that several factors were significantly associated with cognitive deficits. These included having obtained a secondary grade of education (*β* = 0.434), experiencing insomnia (*β* = 0.411), and the interaction between COVID-19 diagnosis and cognition at baseline (*β* = 0.796). These factors were found to be strongly linked to a greater cognitive deficit as illustrated in Table 4. However, no significant associations were found between global cognition and clinical symptoms, autonomy, or depression (*p* > 0.05). We have also estimated the effect size of the study variables to assess the practical significance of the value of p. A value of 0.01 indicates a small effect, a value of 0.06 indicates an effect of moderate size whereas an effect of 0.14 indicates a large effect size. This suggests that these factors did not significantly impact cognitive functioning after the COVID-19 contagion. It is important to note that these findings are specific to global cognition and may not necessarily apply to other cognitive domains or measures.

**Table 4:** General linear model univariate analysis using global cognition as the dependent variable.

Study variables	*ß* (95%CI)	Effect size (n^2^)	Value of *p*
Age	−0.043(−0.169,0.699)	0.029	0.231
Gender (f vs. m)	0.210(−0.621,0.670)	0.032	0.521
BMI			
Educational (primary vs. middle)	0.398(−0.722,0.811)	0.026	0.313
Education (secondary vs. university)	0.434(−0.428,0.919)	0.055	0.032
PANSS (positive)	−0.023(−0.861,0.225)	0.000	0.422
PANSS (negative)	−0.031(−0.082,0.065)	0.004	0.610
PANSS (general psychopathology)	−0.043(−0.092,0.058)	0.002	0.734
CDSS (depression)	0.031(−0.090,0.988)	0.000	0.890
A cognitive assessment at baseline	0.043(−0.523,0.720)	0.003	0.899
The duration between cognitive assessments	−0.138(−0.487,0.342)	0.002	0.602
Chlorpromazine equivalent dose	−0.078(−0.498,0.879)	0.023	0.323
ADS	−0.071(−0.199,0.788)	0.043	0.299
ADL	−0.001(−0.870,0.850)	0.001	0.910
Insomnia	−0.411(−0.273,0.550)	0.026	0.021
The interaction between COVID-199 diagnosis and cognition at baseline.	0.796(−0.233,0.999)	0.198	0.04

Significant level *p* < 0.05.

## Discussion

The present research presents a unique analysis of cognitive fluctuations in individuals diagnosed with schizophrenia, both with and without COVID-19. The results indicate that COVID-19-diagnosed patients with schizophrenia who contract severe acute respiratory syndrome coronavirus-2(SARS-CoV-2) experience significant cognitive decline in working memory, motor speed, attention, information processing speed, and sleep. The study also demonstrated that COVID-19 broadly impacts cognitive function in all patients, suggesting a widespread effect on cognitive abilities. These cognitive processes are essential for language processing, communication, and temporary information storage and manipulation. Working memory and Verbal memory are particularly affected, with COVID-19-diagnosed patients displaying more inadequacy in these domains compared to those without COVID-19. These findings suggest that COVID-19 may have specific effects on memory processes in individuals with schizophrenia. These results are consistent with previous research on cognitive impairments in patients with schizophrenia and highlight the exacerbating potential impact of viral infections on cognitive function (32, 33). It is worth noting that Individuals diagnosed with schizophrenia are more vulnerable to COVID-19 infection compared to the general population. Viral infections, including COVID-19, can intrude on the central nervous system and impact both the cardiovascular and respiratory systems as well as neurological functioning. These infections have been associated with delayed neurodevelopment, various neurological complications, and a decline in the cognitive performance field (34).

Moreover, variations in cognitive impairment across different studies may be attributed to methodological differences, such as variations in cognitive assessment scales and characteristics of the study population. Patients suffering from COVID-19 acute respiratory distress syndrome often encounter cognitive impairment, both in immediate and prolonged scenarios (35, 36). Similarly, experimental research has indicated that infections from the Zika virus can lead to enduring impacts on neurodevelopment, such as cognitive impairment (37). Regarding COVID-19 specifically, recent investigations have revealed cognitive impairment, especially in the domain of attention, among recovered individuals. (10, 38). Another study in Denmark found that a notable proportion of individuals who have recovered from COVID-19 experience cognitive impairment, particularly in verbal learning and executive functions, within three to 4 months after being discharged from the hospital field (39).

Furthermore, research has shown that a considerable proportion of people with moderate or asymptomatic COVID-19 have a cognitive impairment, particularly in the attention and executive function field (40). Uncertainty exists regarding the processes causing the cognitive impairment seen in COVID-19 individuals. The virus may, however, directly infiltrate the central nervous system and cause brain damage (41). Additionally, the inflammatory response triggered by the virus, including the cytokines disruption, can accelerate neurological changes and disrupt neural links, thereby affecting executive function, attention, and memory field (5). In this research, working memory, attention, and motor speed were portrayed as the cognitive skills that were most severely disrupted among COVID-19 patients. A recent comprehensive analysis of recovered COVID-19 patients revealed a rise in inflammatory markers that are known to have substantial impacts on working memory and attention field (14). Working memory, a crucial cognitive function for temporary information storage and manipulation, appears to be significantly impaired among patients diagnosed with schizophrenia who have also contracted COVID-19. This suggests that the viral infection may worsen existing cognitive deficits in this population. It has been suggested that the inflammatory response triggered by viral infections, including COVID-19, may contribute to cognitive decline through mechanisms such as neuroinflammation and oxidative stress (42).

The significant declension in motor tasks observed in individuals with COVID-19 can be attributed to the inherent characteristics of schizophrenia, such as slowed movements and decreased psychomotor activity. This is not only evident in our sample but is also corroborated by additional research. Mistry et al. (43) identified substantial deficits in motor coordination and fine motor skills in Individuals diagnosed with schizophrenia compared to individuals without the condition (44). A similar study by Wood et al. (44) reported significant motor impairments, including reduced manual dexterity and coordination, in patients with schizophrenia (45). Furthermore, the role of neuroleptic treatment in influencing certain aspects of psychomotor performance cannot be overlooked (46). This point is further emphasized in a research investigation by Zhuo et al. (46), which found that certain antipsychotic medications were associated with worsened motor functioning in individuals diagnosed with schizophrenia (38, 47). These consistent findings underscore the prevalence of motor deficits in schizophrenic individuals.

According to our research findings, both groups demonstrated a significant melioration in verbal memory following the COVID-19 period. This improvement can be attributed to administering remediation treatment or antipsychotic medications. These findings align with a separate cohort study by Tensforde et al. (47), which also reported a notable enhancement in verbal memory in schizophrenic patients following the onset of COVID-19 (47). Additionally, another meta-analysis examined the impact of antipsychotic medications on cognitive abilities and found that certain antipsychotic drugs were linked with improvements in verbal memory, supporting our findings (48). Contrary to our findings, a study by Lin et al. (49) reported no substantial spike in verbal memory in patients with schizophrenia following the COVID-19 period (49). This discrepancy may be due to differences in sample size, treatment approaches, or other factors that should have been accounted for in our study. To obtain a more comprehensive understanding of the factors that impact verbal memory in individuals diagnosed with chronic schizophrenia, it is necessary to conduct additional research using larger sample sizes and controlled experimental designs. Additionally, exploring the potential impacts of anti-psychotic medications on cognitive function over an extended period in this population would provide valuable insights.

It is pertinent to emphasize that there were no significant changes in the prevalence of depression among patients before and after COVID-19. This suggests that the individuals in our study did not have depressive thoughts or psychological reactions specifically related to the virus. It is possible that patients living in a community setting did not face the same level of anxiety and stress associated with the COVID-19 pandemic. Previous studies have established significant associations between viral illnesses like varicella-zoster virus, herpes simplex virus, hepatitis C, influenza A(H1N1), HIV/AIDS, and the development of anxiety and depression (50). These findings suggest that viral infections can hurt mental health. Concerning our study, however, it is crucial to acknowledge that the effects of viral infections on mental health can vary based on the specific virus and individual factors. A study found that individuals infected with the varicella-zoster virus had a high risk of developing depression in comparison to non-infected individuals (51).

Similarly, a survey claimed a monumental association between HIV/AIDS infection and increased rates of anxiety and depression (52). Therefore, while our research did not observe a substantial association between the period of COVID-19 and depression among individuals diagnosed with schizophrenia, it is crucial to acknowledge that viral infections can have diverse effects on mental health. Additional investigation is warranted to delve into the precise mechanisms that connect viral infections and mental well-being outcomes in individuals with persistent schizophrenia.

Furthermore, our results revealed that the patient’s daily functioning remained stable and unaffected before and after COVID-19. This phenomenon may be attributed to individuals with schizophrenia who reside in clinical settings often experiencing restricted community engagement and occupational performance opportunities, leading to a more sedentary lifestyle. It is worth mentioning that there was no notable alteration in the utilization of antipsychotic medications before and during the COVID-19 period. These results concord with a study by Anderson et al. (2023), which reported that individuals with schizophrenia who maintained regular medication adherence had a reduced risk of COVID-19 infection and demonstrated better overall health outcomes (9). Previous findings also revealed that individuals with psychiatric conditions who adhere to their medication regimen have a lower chance of getting COVID-19 and tend to experience more favorable outcomes if they become infected compared to the general population (53). Additionally, a comprehensive analysis by Brown et al. (8) highlighted that anti-psychotic medication adherence was associated with improved clinical outcomes and reduced hospitalizations in patients with schizophrenia (54).

The regression analysis results suggest that insomnia and the relationship of a COVID-19 diagnosis with preexisting cognitive function predict cognitive function following the COVID-19 period. This interaction may be attributed to the immune-inflammatory response caused by the virus. Several studies have provided evidence supporting the role of immune-inflammatory processes in cognitive impairments across various neuropsychiatric disorders (55, 56). Inflammation has been identified as a significant neuropsychological process contributing to cognitive decline. Some studies support the notion that COVID-19 can lead to cognitive impairments; other studies have reported conflicting findings. For instance, a recent study found no significant association between COVID-19 and cognitive function in individuals with schizophrenia (3).

Similarly, a meta-analysis reported mixed results regarding the consequence of COVID-19 on cognitive function, with some studies showing impairments and others showing no significant effects (4). Research has shown that low-grade inflammation is intertwined with cognitive impairment in individuals with schizophrenia (1). Furthermore, chronic insomnia has been linked to impairments in attention, memory, executive function, and overall cognitive performance (57). Hence, the coexistence of insomnia and the interplay between a COVID-19 diagnosis and pre-existing cognitive function could contribute to the cognitive impairments observed in individuals following the COVID-19 period. These contrasting findings emphasized the need for more research to understand better the relationship between COVID-19 cognitive function and insomnia in individuals with schizophrenia.

In summary, the available evidence indicates that insomnia and the interplay between a COVID-19 diagnosis and pre-existing cognitive function may potentially impact cognitive abilities in individuals with schizophrenia following the COVID-19 period. However, it is essential to note that the existing literature presents conflicting results. Therefore, further study is requisite to establish a more evident apprehension of the connection between COVID-19, insomnia, and cognitive function in this specific population. Additionally, future studies should uncover the underlying mechanisms contributing to these associations.

### Limitations

It is important to acknowledge several limitations of the study that should be taken into account when interpreting the findings. Firstly, the relatively small sample size of 219 patients may limit the generalization of the results to a larger population of individuals with schizophrenia. Additionally, the study was conducted in a specific cultural and geographical context (Mayo Hospital, Lahore, Pakistan), which introduces potential selection bias and restricts the applicability of the findings to other healthcare settings. Furthermore, the follow-up period of approximately 6.52 ± 1.94 months may not be long enough to capture the long-lasting impact of COVID-19 on cognitive functions in individuals with schizophrenia. Lastly, the absence of a control group of individuals without schizophrenia makes it arduous to isolate the specific impact of COVID-19 on cognitive changes in this population.

## Conclusion

The findings of our investigation indicate a significant decrease in objective cognitive functioning among individuals diagnosed with both schizophrenia and COVID-19. The transmission of the virus has been observed to exert a discernible impact on various cognitive facets, encompassing verbal memory, working memory, and overall cognition. Individuals diagnosed with COVID-19 demonstrated significantly more prominent impairments in these areas compared to those individuals who were not infected. Insomnia has emerged as the predominant determinant in predicting cognitive deterioration, compounded by the interaction of a COVID-19 diagnosis and existing global cognitive functioning. Additional investigation is required to acquire a comprehensive comprehension of the cognitive disparities observed in individuals diagnosed with schizophrenia who have been impacted by the COVID-19 virus.

## Data availability statement

The original contributions presented in the study are available upon reasonable request. Further inquiries can be directed to the corresponding authors.

## Ethics statement

The studies involving humans were approved and conducted by the guidelines of the Declaration of Helsinki and the Ethics Review Committee of Mayo Hospital, Lahore, Pakistan (approval: MH-98975/29). The studies were conducted in accordance with the local legislation and institutional requirements. The participants provided their written informed consent to participate in this study.

## Author contributions

AM: Conceptualization, Formal analysis, Investigation, Methodology, Writing – original draft. ALA: Conceptualization, Investigation, Methodology, Resources, Validation, Writing – review & editing. ABA: Conceptualization, Data curation, Funding acquisition, Investigation, Project administration, Writing – review & editing. FZ: Investigation, Methodology, Resources, Supervision, Validation, Writing – review & editing. IA: Writing – review & editing. BS: Conceptualization, Investigation, Resources, Supervision, Validation, Writing – review & editing.
